# Older Aboriginal and Torres Strait Islander populations living with dementia: State and territory scenario‐based projections into the future

**DOI:** 10.1111/ajag.70068

**Published:** 2025-07-23

**Authors:** Joanne Luke, Jeromey Temple, Tom Wilson, Ruth Williams, Sean Taylor, Dina LoGiudice

**Affiliations:** ^1^ Demography, Centre for Health Policy, School of Population and Global Health, Faculty of Medicine, Dentistry and Health Sciences The University of Melbourne Melbourne Victoria Australia; ^2^ Onemda: Aboriginal and Torres Strait Islander Health and Wellbeing Unit, School of Population and Global Health & Melbourne School of Health Science, Faculty of Medicine, Dentistry and Health Sciences The University of Melbourne Melbourne Victoria Australia; ^3^ Department of Medicine and Aged Care The Royal Melbourne Hospital Parkville Victoria Australia

**Keywords:** ageing, Australian Aboriginal and Torres Strait Islander peoples, dementia, Indigenous peoples

## Abstract

**Objective:**

To produce plausible estimates of the number of Aboriginal and/or Torres Strait Islander people living with dementia within Australia disaggregated by state and territory to mid‐century.

**Methods:**

The Wilson–Grossman variation of the Hamilton–Perry projection model was used to prepare state and territory projections of the Aboriginal and/or Torres Strait Islander population, 2021–2051. Data from the Australian Bureau of Statistics Census (2021) and Australian Institute of Health and Welfare 2021 were used to estimate the number of people living with dementia 2021–2051.

**Results:**

From 2021 to 2051, the Australian Aboriginal and/or Torres Strait Islander population is projected to grow across all states and territories. By 2051, there will be an increasing number and proportion of the population aged over 45, 65 and 80 years, with this increase varying by jurisdiction. This pattern of ageing will see an increase in the number of Aboriginal and/or Torres Strait Islander people living with dementia, regardless of assumptions about future dementia prevalence. This increase will vary in magnitude by juristiction, doubling in the Northern Territory and increasing more than fivefold in the Australian Capital Terrritory, New South Wales, Tasmania and Queensland. Sensitivity analyses of alternative dementia scenarios produce highly similar results.

**Conclusions:**

Between 2021 and 2051, the estimated number of Aboriginal and/or Torres Strait Islander people living with dementia is projected to increase. Combined with regional demographic variations, this trend highlights the urgent need for targeted responses at both local and national levels.

**Policy Impact**

This study found that the Aboriginal and Torres Strait Islander population living with dementia will increase by mid‐century across all Australian states and territories. Our findings highlight the importance of having spatial data at the regional level to effectively plan health, social and aged care for this diverse population.

## 1 INTRODUCTION

Dementia is a leading cause of death in Australia, as in many countries worldwide.^1^ At a time when many populations are ageing, an understanding of the prevalence, drivers and futures of people living with dementia is critical, not only from an epidemiological perspective but also to inform the future planning and provision of medical, social and aged care services to this growing population.^2^ For Aboriginal and Torre Strait Islander populations, Australian government policy frameworks highlight the importance of planning for and current limitations with existing health‐care provision and services.^3^, ^4^

The Australian Institute of Health and Welfare (AIHW) projects that the number of people living with dementia will increase twofold by mid‐century.^5^ However, there remains a paucity of spatial information and modelling on the prevalence of dementia now and into the future among Aboriginal and Torres Strait Islander people.^6^ The three most recent dementia cohort studies involving Aboriginal and Torres Strait Islander populations have provided a strong evidence base to suggest that prevalence rates of dementia are up to 3–5 times higher than in non‐Indigenous Australians.^7^, ^8^, ^9^ Recent national level modelling indicates that the population of older Aboriginal and Torres Strait Islander people living with dementia is projected to increase significantly by mid‐century, driven in part by population growth and ageing.^10^ Further, recent national estimates suggest that the population of older Aboriginal and Torres Strait Islander people aged 45 years and older will increase by nearly 170% by mid‐century. Notably, the population aged 85 years and over is projected to grow at an even faster rate, highlighting the need for age‐specific planning and support.^11^

Despite this recent research and the implications for service planning and provision, no study has considered the future ageing of Aboriginal and Torres Strait Islander people living with dementia across Australia's states and territories. A geographic understanding of dementia futures is important for health‐care provision, caring and planning. Our paper seeks to address this gap by (i) examining structural and numeric ageing of the Aboriginal and Torres Strait Islander population in Australian states and territories from 2021 to 2051, and (ii) understanding the growth of this population living with dementia under various prevalence scenarios.

## 2 METHODS

### 2.1 Ethical considerations

Ethics approval for this study was granted by the University of Melbourne Human Research Ethics Committee (Approval number: 2023‐25631‐42542‐4).

This research was conducted by Aboriginal, Torres Strait Islander and non‐Indigenous researchers in accordance with NHMRC guidelines^12^ and corresponds with priorities identified by Aboriginal and Torres Strait Islander communities.^13^

The Hamilton–Perry cohort projection model was used to prepare state and territory projections of the Aboriginal and/or Torres Strait Islander population by sex and age group from 2021 to 2051.^14^, ^15^ As described elsewhere,^16^ this is a simplified model that requires minimal input data and is therefore well‐suited to situations in which the necessary data for standard projection techniques are unavailable or unreliable. In the case of the Aboriginal and/or Torres Strait Islander population, only some demographic data are needed to prepare projections when a standard multistate cohort‐component model is available, and there are quality limitations affecting available births, deaths and migration data. For a comparison of methods used to project the Aboriginal population under different data environments, readers are directed to Wilson et al.^16^

Specifically, the Hamilton–Perry model projects a cohort population forward in 5‐year projection intervals by adding or subtracting cohort population change—using Cohort Change Ratios (CCRs) and Cohort Change Differences (CCDs). Projection calculations are repeated for as many 5‐year projection intervals as required, with projected populations from one interval becoming the start‐of‐interval populations for the next interval.

Projections of the number of people living with dementia were calculated by applying dementia prevalence rates to projected populations by sex and age group. Dementia prevalence rates for Aboriginal and/or Torres Strait Islander people were estimated from national prevalence rates for 2021 published by the AIHW^5^ and 2021 Australian Bureau of Statistics (ABS) Census counts of people reporting dementia.^17^ Due to uncertainty surrounding the true levels of dementia in the population, we estimated two sets of prevalence rates. The first set is based on the national AIHW estimates, with the census data used to estimate ratios to national rates. Because of noisy patterns in rates at young and middle adult ages due to relatively few people with dementia, the Census‐based ratios were only calculated from dementia amongst the population aged over 75 years.

The second set of prevalence rates is based on Census counts of dementia. Smoothing over age was again applied due to small numbers. We took the census‐based rates for the national Aboriginal and/or Torres Strait Islander population and applied the ratio of Aboriginal and/or Torres Strait Islander state rates for ages over 75 years to the national Indigenous rates for ages over 75 years.

#### 2.1.1 Input data and projection assumptions

Aboriginal and/or Torres Strait Islander Estimated Resident Populations (ERPs) by sex and 5‐year age group for 2011, 2016 and 2021 were obtained from the ABS.^16^, ^17^, ^18^, ^19^, ^20^ Cohort Change Ratios and CCDs were calculated for both 2011–2016 and 2016–2021 periods, averaged over the two periods and smoothed across age to reduce noise.

For both sets of dementia prevalence rates, three future scenarios were formulated. These were as follows:

- Constant: Prevalence rates estimated for 2021 were assumed to remain unchanged.
- Decreasing: Rates were assumed to decline by 1% per year.
- Increasing: Rates increase by 1% per year.

These scenarios reflect considerable uncertainty about the future trajectory of dementia prevalence among the Aboriginal and/or Torres Strait Islander population. Some international studies report declines in prevalence over time,^21^, ^22^ though a few reported increases.^23^

## 3 RESULTS

### 3.1 Aboriginal and/or Torres Strait Islander population projections, 2021–2051

Table 1 displays projected indices of numerical and structural population ageing to mid‐century, including a breakdown by age. Baseline estimates for 2021 reveal that approximately one in five Aboriginal and/or Torres Strait Islander people living in Australia were aged over 45 years, with a majority of those living across the states of New South Wales (NSW) (35%), Queensland (QLD) (27%) and Western Australia (WA) (12%). The age profile of the population varies by state, with Tasmania (TAS) having a slightly older population structure and the Australian Capital Territory (ACT) the youngest, while the Northern Territory (NT) had the smallest percentage of population over 80 years at 1%. Nationally, only 5% of the Aboriginal and/or Torres Strait Islander population was over 65 years and 1% over 80 years.

**TABLE 1:** Aboriginal and/or Torres Strait Islander population count by state and territory, 2021–2051.

	NSW	VIC	QLD	SA	WA	TAS	NT	ACT	AUST
2021									
Population <45	260,817	61,235	212,388	40,518	93,472	25,120	59,006	7627	760,183
Population 45+	78,729	17,463	60,836	11,565	26,565	8774	17,730	1917	223,579
Population 65+	20,138	4313	14,273	2612	5675	2473	3416	401	53,301
Population 80+	2878	587	1831	362	722	345	411	60	7196
Total	339,546	78,698	273,224	52,083	120,037	33,894	76,736	9544	983,762
% <45	77	78	78	78	78	74	77	80	77
% 45+	23	22	22	22	22	26	23	20	23
% 65+	6	6	5	5	5	7	5	4	5
% 80+	1	1	1	1	1	1	1	1	1
2051									
Population <45	661,028	155,704	445,552	82,830	169,914	44,874	59,680	15,032	1,634,614
Population 45+	255,372	61,475	178,615	29,815	70,185	23,684	30,860	8038	658,044
Population 65+	98,530	22,594	67,728	9982	23,553	10,528	8938	3306	245,160
Population 80+	29,089	6258	18,314	2155	5159	3470	1559	1401	67,406
Total	916,400	217,179	624,167	112,645	240,099	68,557	90,540	23,070	2,292,658
% <45	72	72	71	74	71	66	66	65	71
% 45+	28	28	29	27	29	35	34	35	29
% 65+	11	10	11	9	10	15	10	14	11
% 80+	3	3	3	2	2	5	2	6	3
(2021–2051) change									
Population <45	400,211	94,469	233,164	42,312	76,442	19,754	674	7405	874,431
Population 45+	176,643	44,012	117,779	18,250	43,620	14,910	13,130	6121	434,465
Population 65+	78,392	18,281	53,455	7370	17,878	8055	5522	2905	191,859
Population 80+	26,211	5671	16,483	1793	4437	3125	1148	1341	60,210
Total	576,854	138,481	350,943	60,562	120,062	34,663	13,804	13,526	1,308,896
% <45	−5	−6	−6	−4	−7	−9	−11	−15	−6
% 45+	5	6	6	4	7	9	11	15	6
% 65+	5	5	6	4	5	8	5	10	5
% 80+	2	2	2	1	2	4	1	5	2
Population <45G	153	154	110	104	82	79	1	97	115
Population 45 + G	224	252	194	158	164	170	74	319	194
Population 65 + G	389	424	375	282	315	326	162	725	360
Population 80 + G	911	966	900	495	615	906	279	2235	837
Total G	170	176	128	116	100	102	18	142	133

*Note*: G refers to the percentage growth rate over the time period 2021–2051.

Abbreviations: ACT, Australian Capital Territory; AUST, Australia (total across States and Territories); NSW, New South Wales; NT, Northern Territories; QLD, Queensland; SA, South Australia; TAS, Tasmania; VIC, Victoria; WA, Western Australia.

By 2051, there will be a threefold increase in the number of Aboriginal and/or Torres Strait Islander people 45 years and older nationally, owing to numeric ageing of the population. This proportional increase will see a 4.6‐fold increase in the population over 65 years and a 9.3‐fold increase in the population over 80 years. The extent and pattern of ageing is projected to vary by region. For example, although the NT and ACT are projected to have the greatest proportional increase in population aged over 45 years, for the NT, only 2% of the population will be over 80 years compared to 6% of the ACT population.

### 3.2 Estimated Aboriginal and/or Torres Strait Islander population living with dementia, 2021

Table 2 provides estimates of the Aboriginal and/or Torres Strait Islander population living with dementia in 2021 under two baseline prevalence rates considered for this study. Results demonstrated that there is poor harmonisation between AIHW and Census models in estimating the number of people living with dementia (6520 vs. 4454), their age (98% over 45‐year vs. 91%) and gender (60% female vs. 54%). However, both did show that the greatest proportion of people living with dementia was from NSW (33%) and QLD (31%). There were also regional differences in sex ratios, with the NT estimating a far greater proportion of women living with dementia with a sex ratio of approximately 3:1.

**TABLE 2:** Estimated Aboriginal and/or Torres Strait Islander population living with dementia, 2021 (AIHW‐ and census‐adjusted scenario).

	NSW	VIC	QLD	SA	WA	TAS	NT	ACT	AUST
AIHW‐adjusted prevalence									
Males	966	157	851	124	250	99	115	15	2576
Females	1215	235	1142	230	587	142	371	23	3944
Total	2181	391	1992	353	837	242	486	38	6520
% AUST total	33	6	31	5	13	4	8	1	100
Total 45+	2135	382	1942	344	813	237	471	36	6361
Total 65+	1823	326	1636	287	680	204	385	30	5371
Total 80+	817	144	687	129	302	90	166	14	2348
% Female	56	60	57	65	70	59	76	60	60
% 45+	98	98	97	97	97	98	97	97	98
% 65+	84	83	82	81	81	84	79	81	82
% 80+	37	37	34	36	36	37	34	38	36
Census‐adjusted prevalence									
Males	753	126	691	100	212	74	101	13	2069
Females	723	141	694	141	357	84	231	14	2385
Total	1476	267	1385	241	569	158	332	27	4454
% AUST total	33	6	31	5	13	4	8	1	100
Total 45+	1356	243	1255	218	512	148	296	24	4052
Total 65+	1029	183	922	160	373	115	210	17	3009
Total 80+	434	76	363	68	154	48	85	8	1234
% Female	49	53	50	58	63	53	70	52	54
% 45+	92	91	91	91	90	93	89	88	91
% 65+	70	69	67	66	66	73	63	63	68
% 80+	29	28	26	28	27	30	26	28	28
Ratio AIHW‐ to census‐adjusted estimates									
Males	1.28	1.25	1.23	1.23	1.18	1.34	1.14	1.16	1.24
Females	1.68	1.67	1.64	1.63	1.65	1.68	1.61	1.62	1.65
Total	1.48	1.47	1.44	1.47	1.47	1.53	1.47	1.40	1.46
Total 45+	1.57	1.58	1.55	1.58	1.59	1.60	1.59	1.53	1.57
Total 65+	1.77	1.79	1.77	1.79	1.82	1.77	1.83	1.78	1.79
Total 80+	1.88	1.90	1.89	1.89	1.96	1.88	1.96	1.87	1.90
% Female	1.14	1.13	1.14	1.11	1.12	1.10	1.10	1.16	1.13
% 45+	1.07	1.07	1.08	1.07	1.08	1.05	1.08	1.10	1.07
% 65+	1.20	1.22	1.23	1.22	1.24	1.16	1.25	1.27	1.22
% 80+	1.27	1.29	1.32	1.29	1.33	1.23	1.34	1.33	1.30

Abbreviations: ACT, Australian Capital Territory; AIHW, Australian Institute of Health and Welfare; AUST, Australia (total across States and Territories); NSW, New South Wales; NT, Northern Territories; QLD, Queensland; SA, South Australia; TAS, Tasmania; VIC, Victoria; WA, Western Australia.

### 3.3 Projections of the Aboriginal and/or Torres Strait Islander people living with dementia, 2051

Table 3 presents the projections for the Aboriginal and/or Torres Strait Islander population living with dementia by 2051, using the AIHW‐adjusted prevalence rates assuming scenarios of a constant, high and low trend in prevalence rates. In all three scenarios, the number of people living with dementia is expected to increase across all states and territories. Under constant trends where the prevalence rates for dementia remain unchanged from 2021 to 2051, the number of Aboriginal and/or Torres Strait Islander people living with dementia will increase 5.9‐fold nationally (from 6520 people to 38,285). Similarly, for estimates under both low and high prevalence trends, there will be a 4.3‐fold and 7.9‐fold increase, respectively. Table 4 replicated these analyses for the Census‐adjusted rates, again assuming a low, constant and high trend in prevalence rates. Under these three scenarios, findings are consistent with AIHW‐adjusted rates projecting a 3.8‐fold, 5.1‐fold and 6.9‐fold increase in the number of Aboriginal and/or Torres Strait Islander people living with dementia.

**TABLE 3:** Projected Aboriginal and/or Torres Strait Islander population living with dementia, 2051 (AIHW‐adjusted).

	NSW	VIC	QLD	SA	WA	TAS	NT	ACT	AUST
Constant									
Males	6051	1168	5241	599	1282	540	270	233	15,383
Females	8316	1490	7125	894	2694	948	1124	310	22,902
Total	14,367	2659	12,366	1493	3976	1488	1394	542	38,285
% AUST total	38	7	32	4	10	4	4	1	100
Total 45+	14,231	2632	12,243	1473	3929	1479	1376	539	37,902
Total 65+	13,372	2450	11,439	1332	3589	1405	1217	518	35,323
Total 80+	8848	1564	7239	734	2112	979	617	414	22,506
% Female	58	56	58	60	68	64	81	57	60
% 45+	99	99	99	99	99	99	99	100	99
% 65+	93	92	93	89	90	94	87	96	92
% 80+	62	59	59	49	53	66	44	76	59
Constant (increment to 2021 constant base)									
Males	5085	1012	4390	476	1032	440	155	218	12,807
Females	7101	1256	5984	664	2107	806	753	287	18,958
Total	12,187	2267	10,374	1140	3139	1246	908	505	31,765
Total 45+	12,096	2249	10,301	1128	3116	1242	906	503	31,541
Total 65+	11,549	2124	9803	1045	2910	1201	833	487	29,952
Total 80+	8030	1420	6552	606	1811	889	451	400	20,158
High (increment to 2021 constant base)									
Males	7202	1420	6224	685	1480	629	249	299	18,189
Females	10,011	1777	8477	977	3049	1138	1146	395	26,970
Total	17,213	3198	14,700	1662	4530	1767	1395	695	45,159
Total 45+	17,075	3170	14,585	1643	4490	1759	1387	692	44,802
Total 65+	16,227	2981	13,806	1511	4166	1692	1259	669	42,310
Total 80+	11,126	1967	9084	863	2550	1231	666	545	28,032
Low (increment to 2021 constant base)									
Males	3517	709	3032	320	700	300	85	157	8820
Females	4946	870	4137	432	1409	560	462	207	13,022
Total	8463	1578	7169	753	2108	861	546	364	21,842
Total 45+	8408	1567	7128	747	2097	859	549	363	21,718
Total 65+	8083	1489	6839	700	1980	837	517	353	20,797
Total 80+	5737	1014	4676	415	1263	635	291	293	14,324

Abbreviations: ACT, Australian Capital Territory; AIHW, Australian Institute of Health and Welfare; AUST, Australia (total across States and Territories); NSW, New South Wales; NT, Northern Territories; QLD, Queensland; SA, South Australia; TAS, Tasmania; VIC, Victoria; WA, Western Australia.

**TABLE 4:** Projected Aboriginal and/or Torres Strait Islander population living with dementia, 2051 (Census‐adjusted).

	NSW	VIC	QLD	SA	WA	TAS	NT	ACT	AUST
Constant									
Males	4086	784	3568	420	913	349	208	141	10,469
Females	4448	822	3856	513	1487	500	645	154	12,424
Total	8534	1606	7424	933	2400	849	852	295	22,893
% AUST total	37	7	32	4	10	4	4	1	100
Total 45+	8201	1540	7126	884	2290	829	813	288	21,973
Total 65+	7306	1355	6295	749	1959	759	670	266	19,359
Total 80+	4572	822	3762	394	1080	503	320	204	11,658
% Female	52	51	52	55	62	59	76	52	54
% 45+	96	96	96	95	95	98	95	98	96
% 65+	86	84	85	80	82	89	79	90	85
% 80+	54	51	51	42	45	59	38	69	51
Constant (increment to 2021 constant base)									
Males	3333	659	2877	320	702	275	107	128	8400
Females	3725	681	3162	373	1130	415	414	140	10,039
Total	7058	1339	6039	693	1831	691	520	268	18,439
Total 45+	6845	1298	5871	666	1778	681	517	264	17,921
Total 65+	6277	1172	5373	589	1586	644	460	249	16,350
Total 80+	4138	746	3400	326	927	456	235	196	10,423
High (increment to 2021 constant base)									
Males	4762	933	4125	467	1021	398	179	177	12,063
Females	5281	968	4511	552	1650	590	639	194	14,385
Total	10,043	1901	8636	1019	2671	988	819	371	26,448
Total 45+	17,853	3310	15,272	1770	4792	1849	1561	705	47,110
Total 65+	17,022	3125	14,519	1638	4473	1781	1433	682	44,672
Total 80+	11,509	2035	9408	923	2698	1273	748	552	29,146
Low (increment to 2021 constant base)									
Males	2274	455	1952	211	465	185	53	91	5687
Females	2572	468	2162	240	744	286	247	100	6819
Total	4846	923	4115	451	1209	471	299	191	12,505
Total 45+	4719	899	4024	437	1185	466	306	190	12,226
Total 65+	4384	821	3741	395	1079	447	286	180	11,333
Total 80+	2953	533	2424	224	647	325	152	143	7402

Abbreviations: ACT, Australian Capital Territory; AUST, Australia (total across States and Territories); NSW, New South Wales; NT, Northern Territories; QLD, Queensland; SA, South Australia; TAS, Tasmania; VIC, Victoria; WA, Western Australia.

At the state and territory level, projections generated using both AIHW and Census‐adjusted prevalence rates reveal that the magnitude of increase in people living with dementia will vary by region, as will the age and sex profiles of the population. Although both prevalence scenarios unanimously show a substantial increase in the number of Aboriginal and/or Torres Strait Islander people living with dementia across all jurisdictions, the NT is expected to have the smallest proportional increase (although still expected to more than double), while the ACT, Victoria (VIC), NSW, TAS and QLD are all projected to have an increase higher than the national (greater than a fivefold increase). Projections for both scenarios showed that by 2051, 70% of the Aboriginal and/or Torres Strait Islander population living with dementia will be from NSW and QLD, up from 64% in 2021.

Disaggregation at the state and territory level showed that despite an overall increase in the age of the population living with dementia across jurisdictions, the structural age of the population living with dementia will differ by jurisdiction, with the ACT's population predominantly over 80 years, while the NT population living with dementia will mostly be younger than 80 years.

## 4 DISCUSSION

A study by Temple et al. (2022) shows there is expected to be considerable growth in the number of Aboriginal and/or Torres Strait Islander people living with dementia in Australia by 2051.^10^ However, this national aggregated data only provide a partial picture of dementia prevalence now and into the future for this culturally and geographically diverse population. A key contribution of this study is that it provides a more detailed spatial description of ageing and the demographic drivers of dementia at a state and territory level, allowing for a more targeted approach to dementia prevention and management.

Data presented here show that regardless of assumptions made about future dementia prevalence, from 2021 to 2051, the number of Aboriginal and/or Torres Strait Islander people living with dementia will more than double across all states and territories owing to the effects of numeric and structural ageing. The magnitude of this increase is expected to vary by state and territory and appears to follow a pattern of life expectancy. For example, the NT, the territory with the youngest age structure and known life expectancy (65.6 years for males and 69.4 years for females), is projected to have the smallest increase in dementia numbers.^24^ Conversely, in the eastern states and territories of the ACT, VIC, QLD, TAS and NSW, where larger proportions of the populations are projected to be over 80 years, there will be a more than fivefold increase in the number of people living with dementia if prevalence rates remain constant.

The patterns and magnitude of structural and numeric ageing across all states and territories project an overall fourfold increase in the population over 65 years, and an eightfold increase in population over 80 years. These demographic shifts mean that the needs of people living with dementia will place increasing pressure on an already overstretched health and aged care system.^25^ We currently know that the Australian health, social, and aged care system is not meeting the current needs of Aboriginal and Torres Strait Islander people with dementia and their families.^26^, ^27^, ^28^ It is a system based on Eurocentric, individualistic and often privatised models of care that were not designed with Aboriginal and Torres Strait Islander populations in mind.^27^, ^29^, ^30^ Given this, many Aboriginal and Torres Strait Islander people have a preference to use Aboriginal and/or Torres Strait Islander community‐controlled organisations or specialist programs and services co‐designed or delivered in partnership with these organisations and communities.^31^, ^32^ The culturally safe, trauma‐informed and holistic model of care underpinning these services is appropriate and acceptable for Aboriginal and/or Torres Strait Islander people.^33^ In recognising this preference, Aboriginal and/or Torres Strait Islander community‐controlled organisations must be adequately resourced to meet needs projected here.

We currently know there are shortages across the Australian health, social and aged care workforce, which has only been exacerbated by the recent COVID‐19 pandemic.^34^ To meet the growing future demand for care for people with dementia, their carers and families, there needs to be resourcing and investment to maintain and build a trained and culturally competent workforce. Increased training and development opportunities to build the Aboriginal and Torres Strait Islander workforce are key to this.^34^, ^35^, ^36^

There is no one‐size‐fits‐all approach to improving dementia care, with access and workforce challenges that are specific to each urban, regional and remote settings. Any broad effort to reduce dementia nationally must also be complemented by co‐ordinated community and state‐based approaches. We know that responding to dementia in Aboriginal and Torres Strait Islander settings is complex given the fragmented nature of the Australian care system, where different components of the health, social and aged care system fall under the responsibility of different commonwealth and state and territory jurisdiction.^25^, ^26^ It is further complicated by the geographic spread and intra‐diversity of the population, which includes populations with additional recognised needs such as the Stolen Generations members.^26^, ^27^ In the coming years, a whole‐of‐service response will be needed to improve the journey of Aboriginal and Torres Strait Islander people as they navigate Australia's health, social and aged care systems. This response must support individuals across the full continuum of care—from prevention, diagnosis and screening to management and end‐of‐life support. These responses should be guided by frameworks for quality and culturally safe care such as the Good Spirit Good Life Framework for healthy ageing for Aboriginal and Torres Strait Islander people.^37^

In terms of dementia prevention, we found that a 10% reduction in the AIHW‐adjusted prevalence rates between 2021 and 2051 could prevent around 10,000 dementia cases (6000 using Census adjusted prevalence rates) amongst Aboriginal and Torres Strait Islander people. This potential reduction would translate to immeasurable benefits for Aboriginal and Torres Strait Islander families and communities and place fewer demands on Australian health, social and aged care systems. Prevention strategies will also need to focus on strengthening awareness and knowledge not just of those at risk or living with dementia, but the whole community and workforce.^29^ Resources developed by and in collaboration with Indigenous organisations and communities have demonstrated prior effectiveness in raising community awareness about dementia.^38^

To realise a reduction in dementia prevalence requires targeted action to prevent and manage modifiable dementia risk factors specific to this population. These include those outlined in the 2020 Lancet Commission on Dementia Prevention, Intervention and Care report^39^ of obesity, physical inactivity, smoking, low education, diabetes mellitus, hypertension, depression, hearing impairment, alcohol consumption, social isolation and traumatic brain injury. These World Health Organization identified risks have been found to attribute to 35% of dementia burden for a Torres Strait Islander population.^40^, ^41^ The evidence base on dementia prevention underscores the importance of responding to modifiable ‘risks’ across the life‐course, which in the Australian and international context includes consideration of the impacts of ongoing colonisation and structuralised racism.^33^, ^42^, ^43^ However, it must be acknowledged that the biggest risk of ‘age’ is non‐modifiable, so a vast majority of dementia projected for this population just cannot be prevented. Consequently, the coming years are expected to see an increasing demand for health, social and aged care services, and the Australian system must be prepared to meet the cultural, social and emotional well‐being needs of Aboriginal and Torres Strait Islander populations.

### 4.1 Limitations

While the Hamilton–Perry model is simple to implement and frequently produces quality short‐term forecasts, it is important to note a few limitations. These include: being unable to produce projections on births, deaths and migration and identify change, as these processes are not explicitly modelled; inability to formulate precise projection assumptions and scenarios based on those components; difficulties in constraining projections for higher geographies; and conceptual limitations due to demographic processes not being calculated as a function of populations‐at‐risk.

High‐quality data on dementia prevalence is instrumental to supporting evidence based decision‐making.^44^, ^45^ Our modelling revealed discrepancies between using AIHW and Census adjusted prevalence rates models and is illustrative of the practical complexity of determining and documenting dementia prevalence now and into the future.^10^ If we are to better understand dementia prevalence for Aboriginal and Torres Strait Islander populations, then we need to improve data quality in existing systems, establish larger representative cohort studies, and strengthen screening and diagnosis. Screening and diagnosis can be strengthened by the application of culturally appropriate diagnostic tools, increased support to clinicians, and the universal integration of dementia screening into existing tools (e.g. Medicare 715 Health Assessments).^25^, ^45^

The 2021 Census allowed all respondents to select from three response options for the sex question: male, female and non‐binary sex. A limitation of our analysis is that we report sex as a male/female binary owing to limitations relating to underlying ERP (estimated resident population) data used and cell size limitations from the census side.

## 5 CONCLUSIONS

Clearer projections of future dementia pathways for Aboriginal and Torres Strait Islander populations across the states and territories will come when the Australian data environment and collection improves. Until then, this study provides us with population estimate scenarios of the number of Aboriginal and Torres Strait Islander people living with dementia at state and territory level for the years 2021–2051. These scenarios reveal that regardless of dementia prevalence rates applied (AIHW or Census) or conditions (increasing, constant or decreasing prevalence), all scenarios universally project that there will be a substantial increase in the number of Aboriginal and Torres Strait Islander people across Australia living with dementia, and that this will vary in magnitude by region. Consideration of these geographic patterns of dementia at a state and territory level will facilitate more targeted and co‐ordinated prevention and management approaches.

## FUNDING INFORMATION

This research was supported by the Australian Research Council Centre for Excellence in Population Ageing Research (CE1101029) and the National Health and Medical Research Council Call for Research into Healthy Ageing of Aboriginal and Torres Strait Islander Peoples (grant no 1170403).

## CONFLICT OF INTEREST STATEMENT

No conflicts of interest declared.

## Data Availability

Data sharing not applicable to this article as no datasets were generated or analysed during the current study.
